# Crosstalk between Wnt/β-Catenin and NF-κB Signaling Pathway during Inflammation

**DOI:** 10.3389/fimmu.2016.00378

**Published:** 2016-09-22

**Authors:** Bin Ma, Michael O. Hottiger

**Affiliations:** ^1^School of Biomedical Engineering, Shanghai Jiao Tong University, Shanghai, China; ^2^Renji Hospital Clinical Stem Cell Research Center, Shanghai Jiao Tong University School of Medicine, Shanghai, China; ^3^Department of Molecular Mechanisms of Disease, University of Zurich, Zurich, Switzerland

**Keywords:** Wnt, β-catenin, NF-κB, gene expression, signaling pathways

## Abstract

Besides its important role in embryonic development and homeostatic self-renewal in adult tissues, Wnt/β-catenin signaling exerts both anti-inflammatory and proinflammatory functions. This is, at least partially, due to either repressing or enhancing the NF-κB pathway. Similarly, the NF-κB pathway either positively or negatively regulates Wnt/β-catenin signaling. Different components of the two pathways are involved in this crosstalk, forming a complex regulatory network. This review summarizes our current understanding of the molecular mechanisms underlying the cross-regulation between the two pathways and discusses their involvement in inflammation and inflammation-associated diseases such as cancer.

## Introduction

Wnt/β-catenin and nuclear factor kappa-light-chain-enhancer of activated B cells (NF-κB) signaling both are conserved pathways that regulate a variety of biological processes throughout the development and adult lifetime of mammals. Aberrations within these two pathways result in a wide range of pathologies, such as cancer, inflammatory and immune diseases, as well as metabolic diseases (1–4). The Wnt/β-catenin and NF-κB signaling pathways regulate, through independent cascades, the expression of different subsets of target genes controlling cell proliferation, cell survival, and differentiation. In addition to these shared functions, Wnt/β-catenin signaling is crucial for development and tissue regeneration, whereas NF-κB is a key master of inflammation. Recent findings suggest that the two signaling pathways cross-regulate each of their activities and functions.

Crosstalk of signaling pathways extends the functions of individual pathways and results in a more complex regulatory network, inherent to the diversity and homeostasis of biological systems. Wnt/β-catenin pathway components modulate inflammatory and immune responses *via* the interaction with NF-κB (5). Reciprocally, NF-κB also influences the activity of Wnt/β-catenin signaling pathway. Depending on the cellular or tissue context, both positive and negative cross-regulation has been observed. The crosstalk between these two pathways can thus significantly influence the progression of inflammation and cancer. Intensive research has revealed NF-κB signaling as an attractive target for the treatment of inflammatory diseases and inflammation-associated cancer (6–8). Members of the Wnt/β-catenin pathway also serve as potential therapeutic targets for many types of cancer (9). Understanding the molecular basis for the cross-regulation thus helps elucidating the underlying pathophysiological mechanisms for inflammation-involved diseases and for developing more specific and effective therapeutic options against these diseases.

In this review, we summarize the current evidence for both positive and negative regulation of NF-κB-mediated inflammation by Wnt/β-catenin signaling and elaborate on the underlying molecular mechanisms. We also describe the reciprocal regulation of Wnt/β-catenin signaling by the NF-κB pathway and novel models for the cooperation of these two pathways in regulating gene transcription. The major components involved in the cross-regulation are discussed. We sought to describe the complexity of the crosstalk between Wnt/β-catenin and NF-κB signaling to link it with the biological importance for inflammation and cancer, and to discuss its potential impact on the development of new therapeutic options.

## Wnt/β-Catenin Signaling Pathway

**Figure 1:**
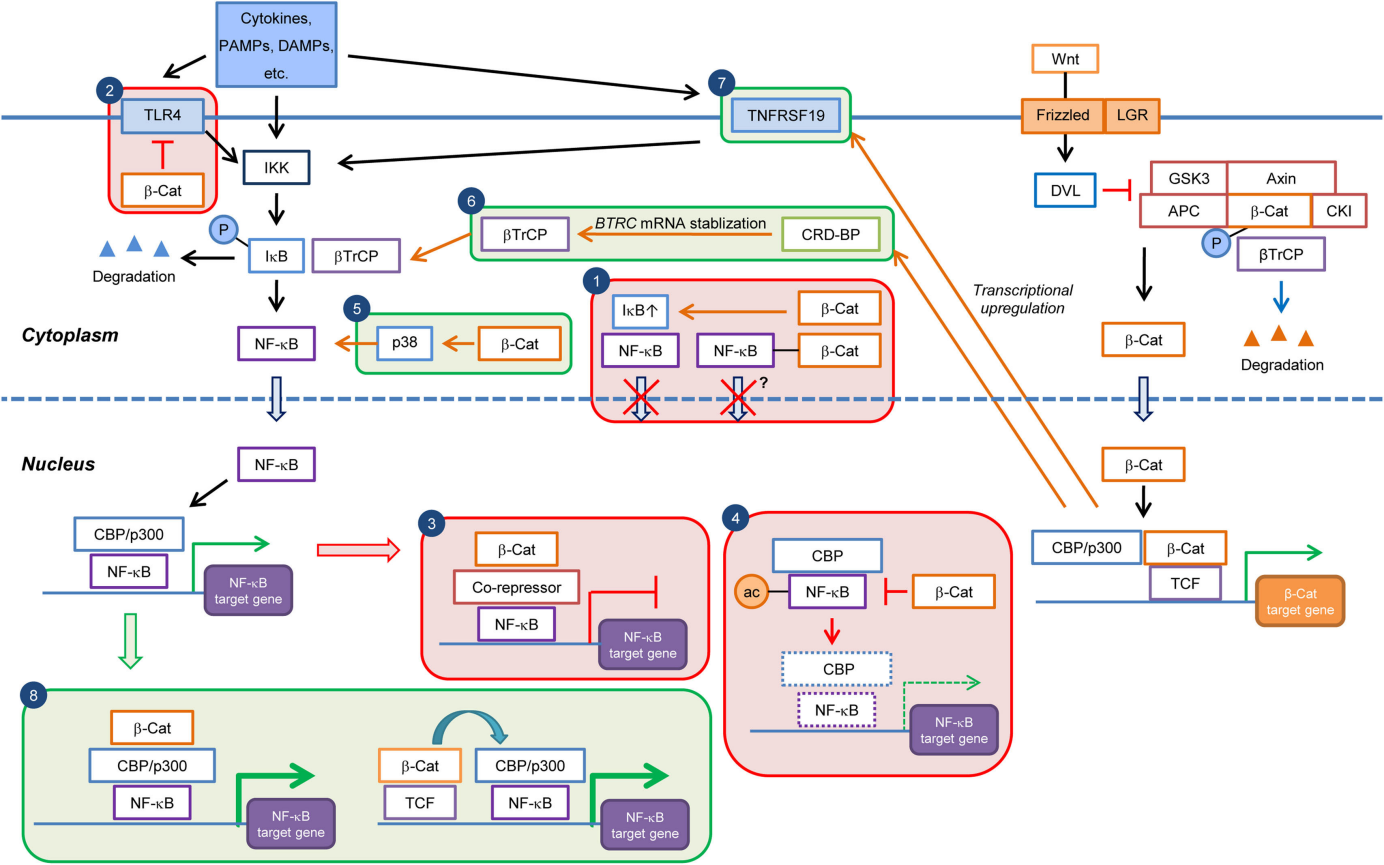
**Current knowledge on the cell type/context-dependent regulation of NF-κB signaling by Wnt/β-catenin pathway**. Boxes in red and green colors indicate negative and positive regulations, respectively. Box 1: inhibition of NF-κB nuclear translocation by β-catenin-mediated sequestration or upregulation of IκB; Box 2: downregulation of TLR4 expression by β-catenin; Box 3: repression of NF-κB target gene expression through recruitment of corepressor at NF-κB-binding elements; Box 4: downregulation of NF-κB target gene expression through inhibition of CBP-mediated acetylation of NF-κB; Box 5: induction of p38 activity and p38-mediated NF-κB activation by β-catenin; Box 6: promotion of βTrCP-mediated IκB degradation through transcriptional upregulation of *CRDBP* by Wnt/β-catenin and CRD-BP-mediated *BTRC* (βTrCP) mRNA stabilization; Box 7: induction of NF-κB activity through transcriptional upregulation of *TNFRSF19* mRNA; and Box 8: enhancement of NF-κB target gene expression through cooperation of β-catenin:TCF/LEF and NF-κB transcriptional complexes. PAMPs, pathogen-associated molecular patterns; DAMPs, danger-associated molecular pattern molecules; β-Cat, β-catenin; P, phospho; ac, acetyl.

Wnt proteins are lipid-modified in the endoplasmic reticulum (ER), traffic through the Golgi to the plasma membrane, and are secreted into the extracellular space (1). Both the human and mouse genome harbors 19 Wnt genes (10). These Wnt proteins are structurally and functionally conserved, and selectively expressed in certain cell types. Extracellular Wnt proteins activate either the β-catenin-dependent, canonical signaling pathway through engagement of the co-receptors frizzled (FZD) and low-density lipoprotein receptor-related protein (LRP) or the β-catenin-independent, non-canonical pathway *via* various receptors such as FZD, receptor tyrosine kinase (Ryk), and receptor tyrosine kinase-like orphan receptor (Ror). β-Catenin is the central mediator of the canonical signaling cascade and functions as an adhesion molecule at the plasma membrane (11). In the absence of Wnt stimulation, β-catenin in the cytoplasm is constitutively targeted for degradation by the destruction complex consisting of adenoma polyposis coli (APC), axin, glycogen synthase kinase 3 (GSK-3), and casein kinase I (CKI) (Figure 1). This complex binds to cytosolic β-catenin and facilitates the latter’s sequential phosphorylation by CKI (at S45) and GSK-3 (at S33/S37/T41). Phosphorylated β-catenin is then recognized and ubiquitinated by β-transducing repeat-containing protein (βTrCP), which tags it for degradation by the proteasome. In the absence of Wnt stimulation, the cytoplasmic levels of β-catenin are thus tightly controlled by this degradation complex. Upon binding of Wnt proteins to the FZD receptor and LRP5/6 co-receptor, the intracellular phosphoprotein disheveled (DVL) is activated, causing the inactivation of the degradation complex and cytoplasmic accumulation of β-catenin. After translocation into the nucleus, β-catenin associates with T cell factor/lymphoid enhancer factor (TCF/LEF) transcription factors and promotes the transcription of its target genes. There are four TCF/LEF transcription factor members in vertebrates: TCF1, TCF3, TCF4, and LEF1. They all contain a β-catenin interaction domain at the N-terminus and recognize the same consensus DNA-binding sequences but are structurally and functionally somewhat different (12). Interestingly, the β-catenin:TCF/LEF machinery not only activates gene expression but also directly represses the transcription of certain target genes (13). Additional co-factors, e.g., cAMP response element-binding protein (CREB)-binding protein (CBP)/p300 (14) and ADP-ribosyltransferase diphtheria toxin-like 1 (ARTD1, also known as PARP1) (15), are involved in the transcriptional regulation of Wnt signaling.

Expression of Wnt/β-catenin signaling target genes regulates stemness (e.g., *NANOG* and *OCT4*), proliferation (e.g., *CCND1* and *MYC*), differentiation (e.g., *SOX9* and *RUNX2*), and immune responses (e.g., *CTLA4* and *TNFSF9*), revealing a broad control of organismal and cellular functions by the Wnt/β-catenin pathway (http://web.stanford.edu/group/nusselab/cgi-bin/wnt/). Aberrant constitutive activation of Wnt/β-catenin signaling caused by mutations in genes, such as *APC* or *CTNNB1* (encoding β-catenin), is involved in tumorigenesis of many organs including intestine, stomach, liver, ovaries, and pancreas (9, 16). Repression of the Wnt pathway by extracellular inhibitors, such as Dickkopf1 (DKK1), Wnt inhibitory factor 1 (WIF1), or secreted frizzled-related proteins (SFRPs), has also been observed in human cancers (16, 17).

Many chemical inhibitors against the Wnt/β-catenin pathway, targeting various components such as porcupine, DVL, tankyrase, or TCF/LEF, have been developed (9). Some reduce Wnt ligand activity and/or the β-catenin protein level, while some directly block transcriptional activity. Wnt/β-catenin signaling has been reported to crosstalk with many other signaling pathways, including NF-κB, mothers against decapentaplegic homolog 3 (Smad3), Notch, forkhead box O (FOXO), and hypoxia-inducible factor-1α (HIF-1α), extending the spectrum of biological functions of this pathway (18–22).

## NF-κB Signaling Pathway

The NF-κB transcription factor family consists of five members trapped in the cytoplasm under non-activated conditions: NF-κB1 (p50/p105), NF-κB2 (p52/p100), RelA (p65), RelB, and c-Rel (23). They all possess a structurally conserved amino-terminal Rel-homology domain (RHD), which contains the dimerization, nuclear localization, and DNA-binding domains. RelA, RelB, and c-Rel (but not p50 or p52) also have a transactivation domain that promotes gene transcription. Upon activation, the NF-κB subunits form either a homo- or heterodimer. NF-κB signaling is divided into the NF-κB essential modulator (NEMO)-dependent, canonical pathway and the NEMO-independent, non-canonical pathway. Various signals, including cytokines, growth factors, microbial products, stress-inducing stimuli such as radiation and oxidative stress, and engagement of T cell receptor (TCR), induce canonical NF-κB signaling (4, 8, 24). These stimuli activate membrane-bound receptors, including the tumor necrosis factor receptor superfamily (TNFRSF) and interleukin-1 receptor (IL-1R)/Toll-like receptor (TLR) superfamily as well as TCR, or intracellular mediators, culminating in the activation of the inhibitor of kappa B (IκB) kinase (IKK) complex, composed of the scaffold protein NEMO (IKKγ) and two IKK subunits (IKKα and IKKβ). The IKK complex subsequently phosphorylates IκB proteins, resulting in their ubiquitination and subsequent proteasomal degradation. As a result, the NF-κB dimers now translocate to the nucleus, where they regulate the expression of target genes. The main activated NF-κB dimers formed in the canonical pathway are RelA/p50 and RelA/p52 (4). Co-factors, such as CBP/p300 and ARTD1, participate in the regulation of NF-κB target gene transcription (25, 26).

By controlling the expression of a broad spectrum of target genes, such as for cytokines, chemokines, growth factors, immune receptors, transcription factors, and repressors of apoptosis, NF-κB functions as a crucial coordinator of inflammatory and immune responses, as well as cell differentiation, proliferation, and survival. Dysregulation of NF-κB has been implicated in diverse inflammatory disorders such as rheumatoid arthritis, multiple sclerosis, asthma, and inflammatory bowel disease (3, 7, 8). NF-κB also regulates cancer development, either through direct control of cell proliferation and apoptosis or induction of cancer-related inflammation and tumor immunity (6, 7). Thus, NF-κB is an attractive therapeutic target for treating inflammatory diseases and cancers. This review focuses on canonical NF-κB signaling, as it is the main cascade that crosstalks with Wnt/β-catenin.

## Regulation of NF-κB Signaling by the Wnt/β-Catenin Pathway

### Negative Regulation of NF-κB Signaling by Wnt/β-Catenin

The first compelling evidence that Wnt/β-catenin negatively regulates NF-κB activity came from the observation that overexpressed β-catenin physically interacted with and inhibited NF-κB function in human colon and breast cancer cells (18) (Table 1). In this study, β-catenin was found to form a complex with RelA and p50, leading to a decrease in NF-κB DNA binding and transactivation activity, and target gene expression. Interestingly, the protein–protein interaction between β-catenin and NF-κB was only indirect, as these two proteins did not bind to each other in an *in vitro* pull down assay using only β-catenin and NF-κB recombinant proteins, while the formation of a complex occurred when cell lysates were supplemented. Activated β-catenin inhibited the expression of the NF-κB target gene *FAS*, thus possibly contributing to tumorigenesis through repression of Fas-mediated apoptosis. A strong inverse correlation between β-catenin and Fas expression was also identified in primary human colon and breast tumor samples, supporting an *in vivo* regulation of NF-κB by β-catenin. Further studies also revealed a negative effect of β-catenin on NF-κB activity and expression of downstream target genes in liver, breast, and colon cancer cells (27, 28). However, such correlation was not seen in head and neck cancer (29), indicating a tissue-specific mechanism and/or more complex regulatory network for these genes. The physical interaction and functional inhibition of NF-κB by β-catenin in colorectal cancer cells was demonstrated to require phosphatidylinositide 3-kinase (PI3K) (30). Blockade of PI3K by chemical inhibitors abrogated the formation of β-catenin and NF-κB protein complexes. In resting colorectal cancer cells, β-catenin and NF-κB colocalized in the cytoplasm, and treatment with PI3K inhibitor resulted in the nuclear translocation of NF-κB and membrane retention of β-catenin. However, it is not clear whether PI3K directly serves as a bridging factor between β-catenin and NF-κB or alternatively plays a role in β-catenin-mediated repression of NF-κB activated by different stimuli.

**Table 1:** **Overview of cell type/context-dependent regulatory effects of Wnt/β-catenin pathway on NF-κB signaling**.

Wnt/β-catenin modulation	Cell types	NF-κB stimulus	Effect on NF-κB signaling	Effect of Wnt/β-catenin on cells	Reference
**Part A. Negative regulation**					
↑ β-Catenin overexpression	Colon and breast cancer cells	TNF-α	Inhibitory physical interaction of β-catenin with NF-κB	Inhibition of Fas expression and cell apoptosis by β-catenin	(18, 30)
↑ β-Catenin overexpression; ↓ APC overexpression	Colon and liver cancer cells	TNF-α; TNF-α + IL-1β + IFNγ	Inhibitory physical interaction of β-catenin with NF-κB	Inhibition of iNos and Fas expression by β-catenin	(27)
↑ GSK3 inhibitor LiCl; ↑ β-catenin overexpression; ↓ β-catenin knockdown	Colon and breast cancer cells	None	Increased NF-κB nuclear accumulation and activation by β-catenin knockdown	Downregulation of uPA/uPAR expression and promotion of cancer cell invasion by β-catenin activation	(28)
↑ Wnt-3A; ↑ GSK3 inhibitor BIO; ↓ β-catenin knockdown	Human chondrocytes	IL-1β	Inhibitory physical interaction of β-catenin with NF-κB	Downregulation of MMP and IL6 expression by β-catenin activation	(31)
↑ Wnt-3A; ↑ β-catenin overexpression	Human fibroblasts	IL-1β; TNF-α	Reduction in CBP-mediated acetylation of RelA by β-catenin	Decrease in expression of cytokines including IL-1β and IL-6 by β-catenin activation	(32)
↑ GSK3 inhibitor LiCl; ↑ mutation-induced stabilization of β-catenin	Intestinal epithelial cells	*Salmonella typhimurium*; TNF-α	Decrease in bacteria- or TNF-α-induced IκB-α degradation and NF-κB activity by β-catenin activation	Reduction in expression of cytokines including IL-6, IL-8, and TNF-α by β-catenin activation	(33, 34)
↑ GSK3 inhibitor SB216763; ↓ β-catenin knockdown	Osteoblasts	LPS	Decrease in IκB-α phosphorylation, RelA nuclear translocation and RelA DNA-binding activity by β-catenin	Repression of CD40 expression and production of cytokines including IL-6, TNF-α, and IL-1β	(35)
↑ Enhanced β-catenin in PTPRO knockout mice	Hepatocytes	Concanavalin A	Attenuated NF-κB nuclear translocation and activation by complexing of β-catenin and NF-κB	Increase in hepatocyte apoptosis and decrease in cytokine secretion in T and NK/NKT cells	(36)
↑ Differential intrinsic β-catenin levels in cell lines; ↑ β-catenin overexpression; ↓ β-catenin knockdown	Prostate cancer	IL-1β	Recruitment of transcriptional corepressors reptin and HDAC to p50 on *KAI* promoter by β-catenin	Downregulation of tumor metastasis suppressor KAI1 and metastatic potential of cancer cells by β-catenin	(39)
↑ GSK3 inhibitor LiCl; ↓ β-catenin knockdown	Diffuse large B-cell lymphoma cells	None	Differential regulations of NF-κB target genes by β-catenin	Suppression of antitumoral CCL3 by activated β-catenin in conjunction with NF-κB	(48)
↓ β-Catenin conditional knockout in mouse liver; ↓ β-catenin knockdown	Hepatocytes	d-galactosamine + LPS; LPS	Enhanced NF-κB activation by dissociation of β-catenin from the RelA/β-catenin complex	Increase in hepatocyte survival and decrease in morbidity and liver injury	(5)
↓ β-Catenin knockdown	Dendritic cells (DCs)	LPS	Upregulation of PTEN/TLR4 expression and NF-κB by β-catenin knockdown	Increase in proinflammatory cytokine programs in DCs and liver ischemia–reperfusion injury by β-catenin knockdown in mice	(40)
**Part B. Positive regulation**					
↑ Overexpression of Wnt-1, Wnt-2, β-catenin, or TCF4; ↓ dnTCF4 overexpression	293T cells, primary human tumors	IKK overexpression	Induction of *BRTC* (βTrCP) mRNA stabilization, βTrCP-mediated IκB-α degradation, and NF-κB activation by β-catenin/TCF	Enhancement of NF-κB-mediated anti-apoptotic function by β-catenin/TCF in cancers	(43, 45)
↑ Overexpression of Wnt-1 or β-catenin; ↓ dnTCF4 overexpression	Vascular smooth muscle cells	TNF-α	Increase of *BRTC* (βTrCP) mRNA level and subsequent NF-κB activation by β-catenin/TCF		(44)
↑ *Apc* conditional knockout in mouse liver	Hepatocytes	LPS	Upregulation of NF-κB activity by β-catenin	Induction of inflammatory program by β-catenin	(51)
↑ Accumulation of β-catenin by E-cadherin loss; ↑ β-catenin overexpression	Melanoma cells	None	Induction of p38 activity and p38-mediated NF-κB activation by β-catenin		(53)
↑ Wnt-1 overexpression; ↓ Wnt-1 knockdown	THP-1 cells	LPS	Increase of RelA protein level by Wnt-1 through induction of SCA expression	Upregulation of inflammatory cytokine and iNOS expression by Wnt-1	(56)
↑ Overexpression of β-catenin or TCF4; ↓ β-catenin knockdown	Colorectal cancer cells	None	β-Catenin/TCF4-mediated transcriptional upregulation of TNFRSF19 which activates NF-κB	Enhancement of NF-κB activity by β-catenin/TCF4	(57)
↑ β-Catenin overexpression; ↓ β-catenin knockdown	293T and HepG2 cells	TNF-α; p50 overexpression	Synergistic interaction between β-catenin and p50 on the promoter of *CRP* gene and enhancement of NF-κB transcriptional activity by β-catenin	Upregulation of CRP expression by β-catenin in association with p50	(49)
↑ LEF1 or β-catenin overexpression; ↓ LEF1 knockdown	Mouse chondrocytes, 293T cells	IL-1β; RelA overexpression	Formation of transcriptional complex consisting of β-catenin/LEF1 and RelA through gene looping on *MMP13* promoter and enhancement of NF-κB transcriptional activity by β-catenin	Enhancement of IL-1β-induced MMP13 expression by β-catenin/LEF1	(50)
↑ TCF4 overexpression; ↓ TCF4 knockdown	Human chondrocytes	IL-1β	Physical interaction of TCF4 with RelA and upregulation of NF-κB activity by TCF4	Increase in MMP and proinflammatory cytokine expression and apoptosis by TCF4	(59)
↑ GSK-3 inhibitor LiCl; ↓ β-catenin knockdown	Diffuse large B-cell lymphoma cells	None	Differential regulations of NF-κB target genes by β-catenin	Augmentation of immunosuppressive IL10 by activated β-catenin in conjunction with NF-κB	(48)
↓ β-Catenin knockdown	Bronchial epithelial cells	LPS	Reduction in NF-κB activity by β-catenin knockdown	Decrease in inflammatory cytokine expression by β-catenin knockdown	(58)

The inhibitory effect of β-catenin on NF-κB activity has also been found in many non-tumor cell types, including chondrocytes (31), fibroblasts (32), epithelial cells (33, 34), osteoblasts (35), and hepatocytes (5, 36) (Table 1). In human chondrocytes, Wnt-3A stimulation or overexpression of Wnt-7B or β-catenin repressed IL-1β-induced expression of *IL6* and matrix metalloproteinase (MMP) genes, including *MMP1, MMP3*, and *MMP13* (31). Physical interaction of β-catenin with RelA and p50 and a reduction in NF-κB reporter activity by Wnt-3A stimulation were observed in this cell type. Notably, a recent study in human fibroblasts showed that IL-1β-induced nuclear translocation of RelA was not directly regulated by Wnt-3A co-stimulation in the initial phase (i.e., 4 h) after NF-κB activation (32). The repression of NF-κB activity by Wnt/β-catenin was rather achieved through a reduction in CBP-mediated acetylation of RelA (Figure 1). The negative effect of Wnt/β-catenin on NF-κB was mediated through CBP, since knockdown of CBP eliminated the downregulation of IL-1β-elicited cytokine expression by Wnt-3A. The selective repression of a subset of proinflammatory cytokine expression by Wnt/β-catenin might be attributed to RelA acetylation-dependent gene expression.

Also in bacteria-colonized intestinal epithelial cells, overexpression of constitutively active mutant β-catenin or activation of β-catenin by GSK-3 inhibition lead to a reduction in NF-κB activity and expression of the target genes *IL6, IL8*, and *TNFA* [encoding tumor necrosis factor-α (TNF-α)], indicating an anti-inflammatory function of β-catenin (33, 34). In line with this notion, lipopolysaccharide (LPS) or concanavalin A (ConA)/TNF-α-induced IκB-α phosphorylation and nuclear translocation of RelA were decreased in osteoblasts treated with GSK-3 inhibitor (35) or in hepatocytes with higher β-catenin levels (36). Moreover, cisplatin- or virus-induced nuclear translocation of RelA was also found to be impaired in cochlea cell line HEI-OC1, monkey kidney MARC-145 cells, and pulmonary alveolar macrophages PAM-CD163 treated with a GSK-3 inhibitor (37, 38). Comparably, bacteria or TNF-α-induced IκB-α degradation was decreased in HCT116 epithelial cells overexpressing constitutively active β-catenin (33) (Figure 1). However, IκB-α was not able to complex with β-catenin in these cells, suggesting that IκB-α stabilization is independent of direct β-catenin binding. It is conceivable that a reduction in IκB-α degradation by β-catenin increases the cytoplasmic retention of NF-κB. The discrepancy of the effect of Wnt/β-catenin on IκB-α degradation and NF-κB nuclear translocation between different studies might be due to different cell types used and/or ways to modulate Wnt/β-catenin and NF-κB signaling. Compared to blocking the overall nuclear translocation of NF-κB, which might influence all target genes, the regulatory effect of β-catenin on RelA post-translational modifications provides a more elaborate and selective control.

In a study of the transcriptional regulation of the tumor metastasis suppressor gene *KAI1* in prostate cancer cells, β-catenin functioned as corepressor, in association with reptin, to inhibit p50-mediated *KAI1* expression (39) (Figure 1). In non-metastatic cells, *KAI1* was actively transcribed by p50 and the transcriptional coactivator Tip60. In metastatic cells where β-catenin was upregulated, β-catenin–reptin complex displaced Tip60, bound to p50 at the *KAI1* promoter and recruited histone deacetylase (HDAC), eventually leading to transcriptional repression. The balance between the transcriptional coactivator and corepressor complexes determined the expression level of *KAI1* and metastatic potential of cancer cells. This cell-type-specific and promoter-specific property underlying β-catenin and NF-κB crosstalk was a unique molecular mechanism for selectively regulating the function of Wnt/β-catenin and might be crucial for cancer metastasis.

A negative regulation of NF-κB signaling by Wnt/β-catenin was also observed in hepatocytes from conditional β-catenin knockout (KO) mice, where lack of β-catenin was found to increase RelA protein levels and LPS-induced NF-κB activation (26). A survival benefit was also observed in d-galactosamine (GalN)/LPS-challenged β-catenin KO mice but not in KO mice with Fas activation, indicating an NF-κB-dependent effect, as the NF-κB-mediated anti-apoptotic effect was enhanced in hepatocytes of GalN/LPS-challenged KO mice, whereas Fas ligand/caspase-mediated apoptosis remained unchanged.

In dendritic cells (DCs), knockdown of β-catenin enhanced TLR4 expression, and LPS-induced NF-κB activation and proinflammatory cytokine programs, providing a potential regulatory mechanism independent of physical interaction of β-catenin with NF-κB (40) (Figure 1). Disruption of β-catenin upregulated phosphatase and tensin homolog deleted on chromosome 10 (PTEN), leading to reduced Akt phosphorylation and Akt-mediated suppression of TLR4. Enhanced TLR4 signaling might thus result in NF-κB activation and increased DC expression of proinflammatory mediators. Elevated inflammatory responses by ablation of β-catenin in DCs were also observed in a mouse model of inflammatory bowel disease, although the dependency of this effect on NF-κB regulation was not investigated (41). In summary, these studies reveal an anti-inflammatory role of β-catenin and that β-catenin signaling programs DCs to a tolerogenic state, limiting the inflammatory response.

These evidences clearly indicate a repressive effect of the Wnt/β-catenin pathway on basal or stimuli-induced NF-κB activity in various cell types *via* different mechanisms. However, the underlying molecular mechanisms seem to be cell type- and/or gene-specific.

### Positive Modulation of NF-κB Activity by Wnt/β-Catenin

While many studies have revealed a negative regulation of NF-κB activity by Wnt/β-catenin, stimulatory effects of Wnt/β-catenin on NF-κB activity have also been reported (Table 1). βTrCP (encoded by the *BTRC* gene) is an E3 ubiquitin ligase receptor targeting the ubiquitination and subsequent degradation of both β-catenin and IκB-α (42). Wnt/β-catenin activation by overexpression of β-catenin or Wnt proteins elevated βTrCP expression, resulting in enhanced degradation of IκB-α and consequently NF-κB transactivation without affecting IKK activity (43). Induction of βTrCP by β-catenin/TCF also accelerated the degradation of β-catenin, serving as an indirect negative feedback loop. In vascular smooth muscle cells, Wnt-1 activated β-catenin/TCF4-mediated βTrCP expression and NF-κB activity (44). Furthermore, it was demonstrated that βTrCP mRNA was stabilized by β-catenin/TCF *via* transcriptional upregulation of its target gene *CRDBP*. Coding region determinant-binding protein (CRD-BP, encoded by the *CRDBP* gene) was found to bind the coding region of βTrCP mRNA, stabilized βTrCP mRNA, and elevated its protein level (45) (Figure 1). Induced *BTRC* and *CRDBP* expression was associated with the activation of both β-catenin and NF-κB in colorectal cancer, implying that integration of these pathways by βTrCP and CRD-BP might contribute to a reduction in tumor cell apoptosis and promotion of tumor metastasis (45, 46). These results suggest that βTrCP and CRD-BP levels are important for coordinating β-catenin/TCF and NF-κB signaling.

It has been demonstrated that stimulation of TLR3 drives breast cancer cells toward a cancer stem cell phenotype, which requires simultaneous activation of the β-catenin and NF-κB signaling pathway, implying a cooperative and synergistic function of β-catenin in TLR3-activated NF-κB signaling (47). A recent study illustrated that the oncogenic mutant scaffold protein CARMA1 causes β-catenin stabilization and connects B-cell receptor (BCR) signaling to NF-κB signaling, thereby coupling β-catenin and NF-κB pathways in diffuse large B-cell lymphomas (48). Increased β-catenin levels were sufficient to induce classical Wnt target gene signatures and additionally were able to augment expression of the immunosuppressive *IL10* and to suppress the antitumoral *CCL3* in conjunction with NF-κB (Figure 1), thus inducing a favorable tumor microenvironment. These data indicate that the effects of β-catenin on NF-κB target gene expression could be gene-dependent in the same cellular context. In line with this notion, downregulation of proinflammatory cytokine expression and synergistic upregulation of adhesion molecule *VCAM1* by Wnt/β-catenin activation were observed in human fibroblasts stimulated with IL-1β (32). Interestingly, a β-catenin:TCF/LEF complex has been shown to bind to promoters of NF-κB target genes, such as *CRP* (C-reactive protein) and *MMP13*, and positively regulate gene transcription through gene looping in concert with NF-κB (49, 50) (Figure 1). In 293T and HepG2 cells treated with TNF-α, both β-catenin and p50 were required for *CRP* expression (49). β-Catenin/TCF4 bound to the *CRP* gene at a site distant to the p50 binding element and physically interacted with p50 through changes in chromosome conformation as detected by 3C chromosome capture assay. The β-catenin/TCF4 complex might constitute the necessary transcriptional machinery, since p50 lacks a transactivation domain. Similarly, in mouse chondrocytes stimulated with IL-1β, β-catenin/LEF1 together with RelA formed a transcriptional complex through gene looping in the *Mmp13* genomic locus to regulate gene transcription (50).

Intriguingly, in contrast to the study in which deletion of β-catenin increased RelA protein levels and target gene expression in hepatocytes from conditional β-catenin KO mice challenged with LPS (5), β-catenin activation in hepatocytes of *Apc* KO mice also potentiated NF-κB activity as judged from electrophoretic mobility shift assay (EMSA) experiments and an inflammatory program including upregulation of NF-κB-induced chemokines (51). These discrepant findings might be explained by β-catenin and *Apc* KO leading to the same effect but by different mechanisms. Although β-catenin and *Apc* KO lead to inactivation and activation of the canonical Wnt pathway, respectively, the elevated NF-κB activity in the liver of *Apc* KO mice might be caused indirectly by tumor-triggered-inflammation, e.g., recruitment of immune cells and subsequent inflammatory responses in tumor microenvironment. Moreover, Wnt/β-catenin signaling-independent functions of APC has also been proposed, such as control of C-terminal binding protein-1 (CtBP1) degradation, retinoic acid (RA) biosynthesis, and cyclooxygenase-2 (COX-2) expression (52), which may as well influence the inflammatory response. In addition, it is possible that the use of different promoter-driven Cre to generate conditional KO in different cell populations might have invariant effects, as the effect of Wnt/β-catenin activation may differ in different subpopulations of cells. In melanoma cells, E-cadherin loss and cytoplasmic β-catenin accumulation triggered p38-mediated NF-κB activation (53). Similarly, E-cadherin disassembly and concomitant GSK-3β inactivation and β-catenin accumulation induced NF-κB-dependent inducible nitric oxide synthase (iNOS) gene expression in hepatocytes (54). Furthermore, E-cadherin in mesenchymal cells formed a complex with β-catenin and RelA and prevented RelA nuclear translocation, thus downregulating fibronectin gene expression, which is positively regulated by both proteins (55). These evidences suggest that E-cadherin is involved in the interaction of β-catenin and NF-κB. In THP-1 cells, stimulation with recombinant Wnt-1 proteins or overexpression of Wnt-1 induced increased RelA protein levels and production of proinflammatory cytokines, including IL-6 and TNF-α, through induction of scavenger receptor A (SRA) expression, which activated NF-κB in response to LPS, although the involvement of β-catenin was not confirmed (56). In another study using colorectal cancer cells, *TNFRSF19* was identified as target gene downstream of the Wnt/β-catenin pathway and TNFRSF19 ligands activated NF-κB signaling, thus revealing an indirect way by which β-catenin positively regulates NF-κB activity (57). In human bronchial epithelial cells, depletion of β-catenin by siRNA reduced LPS-induced NF-κB activation and proinflammatory cytokine expression (58). Overexpression of TCF4 in human chondrocytes augmented NF-κB activity and expression of downstream target genes for several MMPs and proinflammatory cytokines, while LEF1 did not exhibit the same effect (59), suggesting that the synergistic effect of β-catenin and NF-κB transcriptional activity might depend on the TCF/LEF family member depending on the context of genes or cell type. In summary, these studies suggest a potential proinflammatory function of Wnt/β-catenin, which, however, still needs further clarification.

Importantly, the same treatment condition (e.g., with receptor agonists/antagonists or enzyme inhibitors) can influence NF-κB activity differently, depending on whether it is basal or stimulus-induced. For example, it has been shown that hypoxia or prolylhydroxylase inhibition upregulates basal NF-κB activity, while it downregulates IL-1β-induced NF-κB activity in HeLa cells (60, 61). Therefore, although not reported yet, it is possible that under certain conditions, opposing effects of Wnt/β-catenin on NF-κB activity might also be observed between a basal and an induced NF-κB status, or even among different NF-κB-inducing stimuli, as different NF-κB pathway components might be involved under these conditions.

### Control of NF-κB Signaling Activity by GSK-3

While GSK-3 inhibition induced a β-catenin-dependent, negative effect on NF-κB activation and expression of subsets of target genes, β-catenin-independent regulation of NF-κB activity by GSK-3 has also been reported (62, 63). These two branches might be induced simultaneously to regulate the same targets and might as well as control different target genes and biological functions. Overexpression of GSK-3β resulted in repression of TNF-α-induced NF-κB activation, while overexpression of β-catenin had a minimal effect on NF-κB activity in endometrial carcinoma cells (62). GSK-3β overexpression stabilized IκB, thereby inhibiting NF-κB signaling. GSK-3β directly phosphorylated RelA and negatively controlled the basal activity of NF-κB (63). In contrast to the negative role of GSK-3 in NF-κB activation, GSK-3β also positively regulated NF-κB activity without changing IκB degradation or NF-κB nuclear translocation in mouse embryonic fibroblasts (MEFs) (64).

In monocytes treated with TLR agonists, GSK-3 inhibition enhanced CREB-mediated *IL10* expression but repressed the production of NF-κB-mediated proinflammatory cytokines through differentially affecting the interaction of nuclear RelA and CREB with coactivator CBP (65). GSK-3 inhibition increased binding of CBP to CREB but suppressed the binding of CBP to nuclear RelA. In glioma cells, GSK-3 inhibition caused a drastic decrease in NF-κB activity (66). A study in mice also showed that GSK-3 inhibition significantly reduced TLR-mediated chronic intestinal inflammation (67). However, the involvement of β-catenin in the regulatory function of GSK-3 inhibition was not explored in these studies.

It would be interesting to further investigate if different GSK-3 pools are used in Wnt and NF-κB pathways, respectively, upon induction of these pathways and whether also extracellular Wnt-induced signaling regulates NF-κB activity through GSK-3.

### Anti- and Proinflammatory Roles of Wnt/β-Catenin Signaling

Given both repressing and stimulating effects of Wnt/β-catenin signaling on NF-κB activity, it is not surprising that Wnt/β-catenin possesses both anti- and proinflammatory functions at the organismal level. The exact outcome might thus be context-dependent and/or a question of the balance between two contrary functions. So far, most evidence supports that Wnt/β-catenin signaling downregulates production of proinflammatory cytokines, including IL-1β, IL-6, IL-8, and TNF-α, in different cell types and stimulated with various stimuli, such as LPS, cytokines, viruses, and bacteria, regardless of the responsible mechanisms for Wnt/β-catenin-mediated repression of NF-κB activity (31–35, 37, 38).

An anti-inflammatory role of Wnt/β-catenin pathway *in vivo* has been demonstrated in mice (40, 41). In a mouse model of warm liver ischemia and reperfusion injury (IRI), disruption of β-catenin signaling increased hepatocellular damage and enhanced hepatic DC maturation/function and PTEN/TLR4 local inflammation (40). The protective effect of β-catenin was, at least partly, due to induction of PI3K/Akt signaling and a reduced TLR4-driven inflammatory response in DCs. In agreement with this study, another study also points out an anti-inflammatory role of β-catenin by demonstrating that depletion of β-catenin in DCs leads to enhanced inflammatory responses and disease onset in a mouse model of inflammatory bowel disease (41). In addition, GSK-3 inhibition potently suppressed inflammatory responses in mice challenged with LPS (65). However, a cross-regulation between NF-κB and β-catenin in these specific studies has yet to be proven.

Conversely, a proinflammatory role of Wnt/β-catenin pathway *in vivo* has been observed in hepatocellular carcinoma in *Apc* KO mice (51). Oncogenic β-catenin signaling induced an inflammatory program in hepatocytes that involved a direct transcriptional control by β-catenin and activation of the NF-κB pathway. A subset of chemokines was induced by β-catenin activation while none of the canonical proinflammatory cytokines was influenced, implying that oncogenic β-catenin signaling may promote tumor-associated inflammation indirectly through shaping the cellular compositions in the microenvironment. Another study also reported a positive effect of β-catenin in LPS-induced proinflammatory cytokine production in human bronchial epithelial cells (58).

In conclusion, both anti-inflammatory and proinflammatory functions of Wnt/β-catenin pathway were observed, depending on the conditions and regulated through different mechanisms. Wnt/β-catenin also differentially affects NF-κB-mediated subsets of target genes and biological functions (e.g., inflammation, cell proliferation, and apoptosis) in response to different stimuli. It will be crucial to disentangle the precise role of Wnt/β-catenin signaling in inflammation in a cell/tissue- and physiology/pathology-specific context.

## Regulation of Wnt/β-Catenin Signaling by the NF-κB Pathway

Crosstalk between Wnt/β-catenin signaling and the NF-κB pathway not only consists of Wnt/β-catenin regulating NF-κB activity but also the reverse effect of NF-κB on Wnt/β-catenin signaling.

### Negative Regulation of Wnt/β-Catenin Signaling by NF-κB Pathway

**Figure 2:**
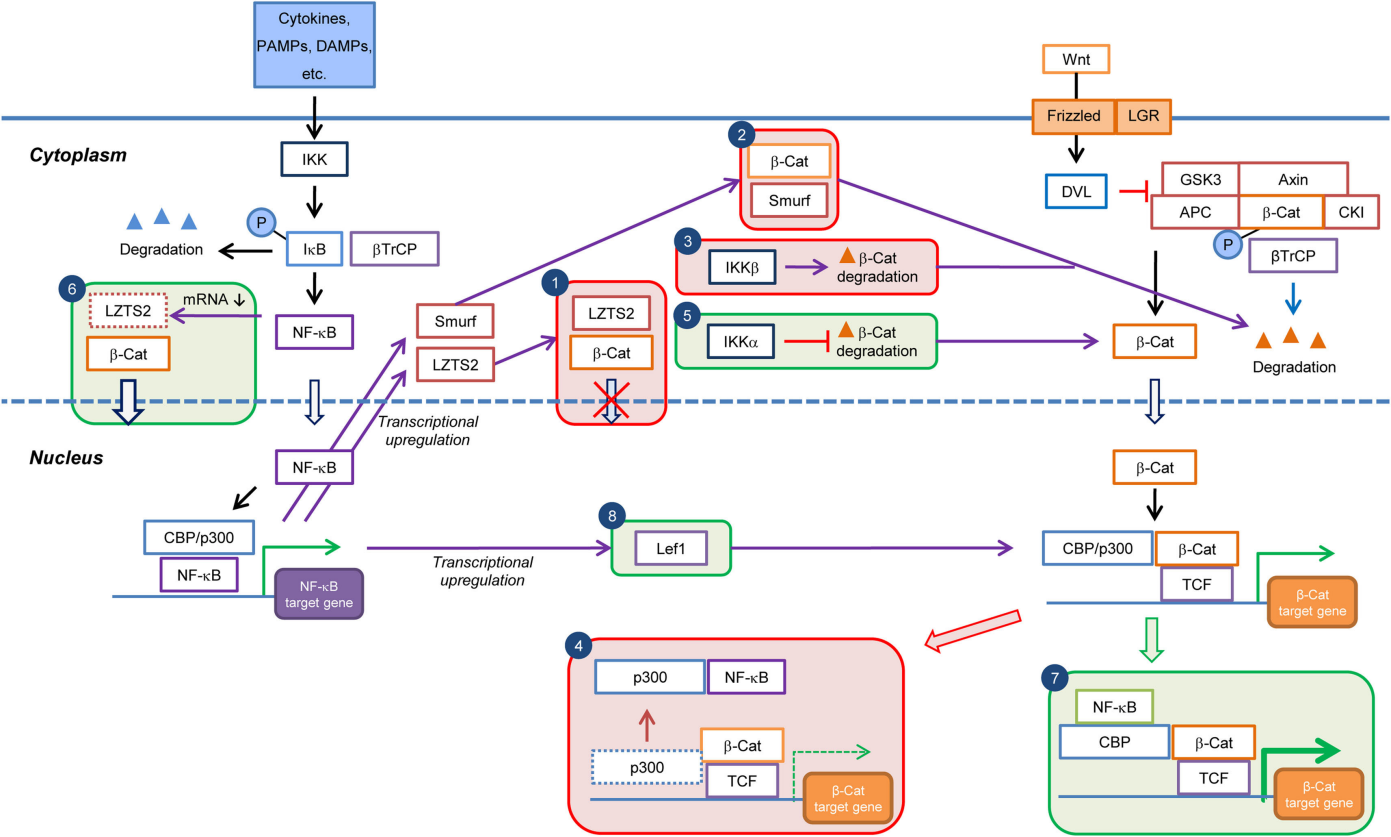
**Current knowledge on the cell type/context-dependent regulation of Wnt/β-catenin signaling by NF-κB pathway**. Boxes in red and green colors indicate negative and positive regulations, respectively. Box 1: inhibition of β-catenin nuclear translocation through NF-κB-induced transcriptional upregulation of *LZTS2*; Box 2: promotion of β-catenin degradation through NF-κB-induced transcriptional upregulation of *Smurf*; Box 3: promotion of β-catenin degradation by IKKβ; Box 4: reduction in Wnt/β-catenin target gene expression through sequestration of transcriptional co-factor p300 from β-catenin by NF-κB; Box 5: inhibition of β-catenin degradation by IKKα; Box 6: increase of β-catenin nuclear translocation by inhibition of *LZTS2* expression by NF-κB; Box 7: enhancement of Wnt/β-catenin target gene expression through cooperation of NF-κB and β-catenin:TCF/LEF transcriptional complexes; and Box 8: enhancement of β-catenin:TCF/LEF transcriptional activity through transcriptional upregulation of *Lef1* expression by NF-κB. PAMPs, pathogen-associated molecular patterns; DAMPs, danger-associated molecular pattern molecules; β-Cat, β-catenin; P, phospho.

Several studies have reported a negative regulation of Wnt/β-catenin by NF-κB (Table 2). NF-κB has been shown to indirectly regulate the Wnt/β-catenin pathway through regulation of target genes that affect β-catenin activity or stability. Leucine zipper tumor suppressor 2 (LZTS2), a putative tumor suppressor, has been shown to interact with β-catenin and inhibit its nuclear localization and transcriptional activity (68). Intriguingly, NF-κB activation was found to inhibit β-catenin/TCF activity through upregulation of *LZTS2* in colon, liver, and breast cancer cells (Figure 2) but downregulate *LZTS2* in glioma cells to promote β-catenin/TCF activity, strongly indicating a cell type-specific effect (69). Furthermore, NF-κB inhibited osteogenic differentiation of mesenchymal stem cells (MSCs) by promoting β-catenin degradation through induction of the E3 ubiquitin protein ligases SMAD ubiquitination regulatory factor 1 (Smurf1) and Smurf2 (70). In colon cancer cells, an extract of *Polysiphonia japonica* downregulated transcriptional activity of Wnt/β-catenin signaling and downstream target gene expression through activation of NF-κB without altering β-catenin levels (71). However, the underlying molecular mechanisms of this finding remain to be elucidated. It is possible that NF-κB activation inhibits the nuclear translocation of β-catenin, as previously described (68), or represses the activity or expression levels of Wnt pathway transcriptional (co-)factors other than β-catenin.

**Table 2:** **Overview of cell type/context-dependent regulatory effects of NF-κB pathway on Wnt/β-catenin signaling**.

NF-κB modulation	Cell types	Wnt/β-catenin modulation	Effect on Wnt/β-catenin signaling	Effect of NF-κB on cells	Reference
**Part A. Negative regulation**					
↑ RelA overexpression	Endometrial carcinoma cells	β-Catenin overexpression	Sequestration of transcriptional co-factor p300 from β-catenin and inhibition of β-catenin-mediated transcriptional activity by RelA		(62)
↑ Extract of *Polysiphonia japonica* (EPJ)	HEK293 cells, colon cancer cells	Wnt-3A; GSK-3 inhibitor LiCl	Inhibition of Wnt/β-catenin transcriptional activity without altering β-catenin protein level by EPJ stimulation	Downregulation of Wnt/β-catenin target gene *CCND1* by EPJ stimulation	(71)
↓ IKKβ small molecule inhibitor IKKVI; ↑ TNF-α; ↑ IL-17	Mesenchymal stem cells (MSCs)	None	Induction of E3 ubiquitin-protein ligases Smurf1 and Smurf2 and promotion of Smurf-mediated degradation of β-catenin by NF-κB activation	Inhibition of osteogenic differentiation of MSCs by NF-κB activation	(70)
↑ RelA overexpression; ↓ overexpression of NF-κB decoy; ↓ NF-κB inhibitor SN50	Colon, liver and breast cancer cells	None	Upregulation of LZTS2, decrease in nuclear translocation of β-catenin, and repression of β-catenin/TCF transcriptional activity by NF-κB		(69)
↓ IKKβ knockout; ↑ IKKβ overexpression	SW480 colon cancer cells, COS-7 kidney cells, MEFs	Overexpression of β-catenin or LEF1	Interaction of β-catenin with IKKβ, phosphorylation of β-catenin, and downregulation of β-catenin protein level by IKKβ		(72, 73)
**Part B. Positive regulation**					
↑ RelA overexpression; ↓ overexpression of NF-κB decoy; ↓ NF-κB inhibitor SN50	Glioma cells, Human adipose tissue- or bone marrow-derived MSCs	None	Downregulation of LZTS2, increase in nuclear translocation of β-catenin, and enhancement of β-catenin/TCF transcriptional activity by NF-κB		(69, 78)
↓ IKKα knockout; ↑ IKKα overexpression	SW480 colon cancer cells, COS-7 kidney cells, MEFs	Overexpression of β-catenin or LEF1	Interaction of β-catenin with IKKα, phosphorylation of β-catenin, and upregulation of β-catenin protein level by IKKα	Upregulation of Wnt/β-catenin target gene *CCND1* by IKKα	(72, 73)
↑ IKKα overexpression	293T cells	Overexpression of β-catenin	Inhibition of both GSK-3β/APC-dependent canonical and SIAH1-mediated non-canonical degradation pathways, and stabilization of cytosolic β-catenin proteins by IKKα		(74)
↓ RelA knockout in mouse intestines; ↑ IκB-α knockout in mouse intestines; ↑ RelA overexpression	Mouse intestinal epithelial cells, 293 cells	β-Catenin stabilization mutation or *Apc* knockout in mouse intestines; overexpression of β-catenin or TCF4	Formation of transcriptional complex of RelA/p50 and β-catenin/TCF through CBP and enhancement of β-catenin/TCF transcriptional activity by NF-κB	Synergistic induction of β-catenin-mediated stem cell signature gene expression and dedifferentiation of non-stem cells by NF-κB activation	(75)
↑ IL-1β; ↓ NF-κB inhibitor SN50	Mouse chondrocytes	None	Direct binding of NF-κB to *Lef1* promoter to induce transcription and enhancement of β-catenin/LEF1 transcriptional activity by NF-κB		(77)
↑ TNF-α; ↓ RelA knockout in mice	Macrophages, lung cancer cells	None	Activation of β-catenin in tumor cells by NF-κB-induced TNF-α from macrophages	Requirement of RelA for cigarette smoke-induced TNF-α production in macrophages and tumor cell growth	(76)

In endometrial carcinoma cells, overexpression of RelA negatively regulated transcriptional activity of β-catenin/TCF (62). In this study, RelA was proposed to inhibit β-catenin-mediated transcription *via* sequestration of the transcriptional coactivator p300 (Figure 2).

In summary, NF-κB signaling negatively regulates Wnt/β-catenin pathway either indirectly through the functions of NF-κB target genes (e.g., *LZTS2* and *SMURF*) or directly by interfering with the formation of transcriptional complex β-catenin/TCF/p300. The direct mechanism may represent a more rapid and instant way to modulate Wnt/β-catenin signaling activity.

### Positive Regulation of Wnt/β-Catenin Signaling by NF-κB Pathway

Several components of the NF-κB signaling pathway, including IKK (72–74) and RelA (75, 76), also seemed to be involved in the positive regulation of Wnt/β-catenin signaling (Table 2). IKKα and IKKβ, the critical activators of the NF-κB pathway, differently regulated β-catenin-dependent transcriptional activity (72). IKKα increased β-catenin turnover, whereas IKKβ had a negative effect on β-catenin levels, although both IKKs interacted with and phosphorylated β-catenin. However, this study did not reveal any conclusive molecular mechanisms for the opposing effects of IKKα and IKKβ on β-catenin protein levels. In agreement with this study, IKKα, but not IKKβ, was found to upregulate β-catenin/TCF transcriptional activity and expression of the downstream target gene *CCND1* (encoding cyclin D1) (73). Furthermore, IKKα stabilized cytosolic β-catenin through inhibition of both the GSK-3β/APC-dependent canonical degradation pathway and seven in absentia homolog 1 (SIAH1)-mediated non-canonical degradation pathway (74) (Figure 2).

A novel role for NF-κB as transcriptional co-factors for β-catenin/TCF has been discovered in the study of dedifferentiation-induced intestinal tumorigenesis in mouse models (75). NF-κB activation potentiated Wnt/β-catenin signaling activity and induced dedifferentiation of non-stem cells that acquired tumor-initiating capacity. The RelA/p50 dimer was bound through CBP to the β-catenin/TCF transcription complex at target gene promoters and augmented the expression of a subset of stem cell signature genes such as *Lgr5, Ascl2*, and *Sox9* (Figure 2).

NF-κB also positively regulated Wnt/β-catenin signaling by affecting expression of Wnt/β-catenin pathway genes (Table 2; Figure 2). In mouse chondrocytes, IL-1β-mediated NF-κB activation induced the expression of the Wnt/β-catenin pathway transcription factor Lef1, presumably the transcriptional activity of β-catenin/LEF, providing an indirect mechanism for the control of Wnt/β-catenin signaling pathway activity by NF-κB (77). In human adipose tissue-derived MSCs and bone marrow stromal cells, activated NF-κB repressed the expression of *LZTS2*, which inhibited nuclear localization and transcriptional activity of β-catenin (78). This is consistent with the observation in glioma but not breast cancer cells or colon and liver tissue, as discussed above (69). Interestingly, the regulation of Wnt/β-catenin signaling by NF-κB can also be observed in between cells. Deletion of RelA largely inhibited cigarette smoke-induced TNF-α production in macrophages as well as TNF-α-mediated β-catenin activation and lung cancer cell growth in mice (76). TNF-α released from macrophages was proposed to activate β-catenin through GSK-3β and Akt signaling in tumor cells, thereby promoting proliferation of the latter.

Evidently, the effects of NF-κB signaling on Wnt/β-catenin activity and the underlying molecular mechanisms are cell type dependent. However, it cannot be excluded that both positive and negative regulations can co-exist in the same cell type, employing different molecular mechanisms, and that the overall observed effect is the sum of two opposing mechanisms.

## Wnt/β-Catenin and NF-κB Crosstalk Links Inflammation with Tumorigenesis

Tumor-promoting inflammation has been recognized as one of the hallmarks of cancer (79). NF-κB serves as a critical link between inflammation and cancer through its ability to upregulate the expression of tumor-promoting cytokines, such as *IL6* and *TNFA*, and survival genes, such as *BCL2L1* (*BCLXL*). Besides the direct control of cell proliferation/survival by NF-κB, the crosstalk between NF-κB and Wnt/β-catenin provides another important intracellular basis for inflammation-induced tumorigenesis. Since Wnt/β-catenin signaling has been shown to be oncogenic in a wide range of tumor types, the positive regulation of Wnt/β-catenin by the NF-κB pathway may contribute to cancer development. A convincing example is the synergistic cooperation between NF-κB and β-catenin/TCF4 on the expression of a subset of Wnt target genes in colon cancer (75). In this model, activated NF-κB functions as a transcriptional coactivator in conjunction with β-catenin/TCF4 to induce a set of stem cell signature genes, such as *Lgr5, Ascl2*, and *Sox9*, and subsequent tumor cell growth.

The Wnt/β-catenin and NF-κB cross-regulation linking inflammation and tumorigenesis not only occur within cells but also in between cells. In a gastric tumor model, *Helicobacter* infection-activated macrophages elicited NF-κB-mediated production of TNF-α, which then inactivated GSK-3β and enhanced the oncogenic Wnt/β-catenin signaling in the gastric cancer cells (80). This model provides a missing link of how *Helicobacter*-induced chronic inflammation prompts oncogenic signaling.

In addition to creating a tumor-favorable microenvironment consisting of various tumor-promoting inflammatory cells (7), NF-κB-mediated inflammation has now been demonstrated to enhance the tumorigenic potential of cancerous cells through upregulation of Wnt/β-catenin signaling, further strengthening the idea that NF-κB may be an attractive therapeutic target for inflammation-associated cancer.

## Mutual Regulation of Wnt/β-Catenin and NF-κB Signaling

In most biological processes, crosstalk of Wnt/β-catenin and NF-κB signaling is bidirectional, meaning that both pathways reciprocally regulate each other. Such mutual regulations are critical for either enforcement or limitation of downstream responses. In a hair follicle development model, Wnt/β-catenin and NF-κB signaling was interdependent for their regulatory activities in hair follicle formation (81). Binding of the A1 isoform of the TNF-α family member ectodysplasin (EDA) and its receptor EDAR induced NF-κB nuclear translocation and activation in developing hair follicle placodes. *Edar* was a direct target of Wnt/β-catenin, and Eda/Edar/NF-κB signaling was required to refine the pattern of Wnt/β-catenin activity and maintain this activity by promoting Wnt10b production at later stages of hair follicle placode development. However, Wnt/β-catenin signaling was initially activated independently of EDA/EDAR/NF-κB activity in primary hair follicle primordia. Maintenance of localized expression of Wnt10b and Wnt10a required NF-κB signaling and Wnt10b was a direct transcriptional target gene of NF-κB, providing a molecular explanation for this observation. Moreover, the Wnt/β-catenin signaling antagonist *DKK4* was a target gene of the EDA/EDAR/NF-κB pathway, serving as a negative feedback to limit β-catenin signaling (82). These data reveal a complex interplay and interdependence of Wnt/β-catenin and NF-κB pathways in the initiation and maintenance of primary hair follicle placodes.

Mutations or expression levels of *APC* and β-catenin levels were found to control the outcome of TLR4 activation by LPS on proliferation of colon cancer cells (83). LPS enhanced cell proliferation in *APC* mutated or depleted cells but inhibited cell proliferation in *APC* wild-type cells. It was proposed that high β-catenin levels blocked NF-κB-mediated cell apoptosis and LPS-induced NF-κB promoted β-catenin expression and β-catenin-regulated proliferation in *APC* mutated or depleted cells where β-catenin degradation was compromised. These data revealed that the reciprocal modulation between β-catenin and NF-κB was involved in the regulation of tumor growth by APC status and TLR4 activation.

Together, the mutual regulation of Wnt/β-catenin and NF-κB signaling is observed in a large number of cell and tissue types and is important for the maintenance of cellular/tissue homeostasis or the reinforcement of cell fate.

## Concluding Remarks

Given the crucial biological functions of the Wnt/β-catenin and NF-κB signaling pathways, it is of particular biomedical interest and importance to understand their crosstalk. A lot of efforts in recent years have revealed a complex network of Wnt/β-catenin and NF-κB signaling interaction. These two pathways are interconnected through physical interactions of mediators and regulation of target genes in the convergence. The regulatory effects are often cell/tissue-dependent or even gene-specific, suggesting that cross-regulation needs to be investigated in a context-oriented fashion. Additional efforts using appropriate cell or animal models are required to understand how the Wnt/β-catenin and NF-κB pathways crosstalk in inflammation and inflammation-associated diseases such as cancer. The further knowledge will eventually help design better interventions for the treatment of inflammatory diseases. When interfering with one of the two pathways, the other pathway might also be changed due to their cross-regulation, thereby potentially causing additional, secondary effects not investigated. It is not just a matter of which molecule to target but also how to interfere with it (e.g., blocking activity without altering the protein level or reducing expression affecting both protein level and activity). For example, when treating cancer with Wnt/β-catenin pathway blockers, one could think about whether this might cause undesired proinflammatory effects due to upregulation of NF-κB activity by elevated β-catenin levels, as hinted from the abovementioned evidence. One potential approach to circumvent the negative feedback is to target the transcriptional activity of β-catenin:TCF/LEF or other coactivators, rather that affecting β-catenin protein turnover. Targeting both pathways simultaneously might also be promising in this case, as well as for other inflammation-associated diseases.

Taken together, the existence of a crosstalk between the Wnt/β-catenin and NF-κB signaling pathways provides new opportunities to treat inflammatory and inflammation-associated diseases such as cancer. Although due to the complex interactions, manipulation of this crosstalk might be challenging to achieve a desired and tissue-specific outcome, our increasing knowledge of mechanisms underlying Wnt/β-catenin and NF-κB signaling cross-regulation will hopefully help resolve these obstacles, so that more effective treatments can be designed.

## Author Contributions

BM and MOH conceived and wrote the manuscript.

## Conflict of Interest Statement

The authors declare that the research was conducted in the absence of any commercial or financial relationships that could be construed as a potential conflict of interest.

## Abbreviations

βTrCP, β-transducing repeat-containing protein; APC, adenoma polyposis coli; ARTD1, ADP-ribosyltransferase diphtheria toxin-like 1; BCR, B-cell receptor; CBP, cAMP response element-binding protein-binding protein; CKI, casein kinase I; ConA, concanavalin A; COX-2, cyclooxygenase-2; CRD-BP, coding region determinant-binding protein; CREB, cAMP response element-binding protein; CRP, C-reactive protein; CtBP1, C-terminal binding protein-1; DCs, dendritic cells; DKK1, Dickkopf1; DVL, disheveled; EDA, ectodysplasin; EDAR, ectodysplasin receptor; ER, endoplasmic reticulum; FOXO, forkhead box O; FZD, frizzled; GalN, d-galactosamine; GSK-3, glycogen synthase kinase 3; HDAC, histone deacetylase; HIF-1α, hypoxia-inducible factor-1α; IκB, inhibitor of kappa B; IKK, inhibitor of kappa B kinase; IL-1β, interleukin-1β; IL-1R, interleukin-1 receptor; iNOS, inducible nitric oxide synthase; KO, knockout; LEF, lymphoid enhancer factor; LPS, lipopolysaccharide; LRP, low-density lipoprotein receptor-related protein; LZTS2, leucine zipper tumor suppressor 2; MEFs, mouse embryonic fibroblasts; MMP, matrix metalloproteinase; MSCs, mesenchymal stem cells; NEMO, NF-κB essential modulator; NF-κB, nuclear factor kappa-light-chain-enhancer of activated B cells; PI3K, phosphatidylinositide 3-kinase; PTEN, phosphatase and tensin homolog deleted on chromosome 10; RA, retinoic acid; RHD, Rel-homology domain; Ror, receptor tyrosine kinase-like orphan receptor; Ryk, receptor tyrosine kinase; SFRPs, secreted frizzled-related proteins; SIAH1, seven in absentia homolog 1; Smad3, mothers against decapentaplegic homolog 3; Smurf, SMAD ubiquitination regulatory factor; SRA, scavenger receptor A; TCF, T cell factor; TCR, T cell receptor; TLR, Toll-like receptor; TNF-α, tumor necrosis factor-α; TNFRSF, tumor necrosis factor receptor superfamily; WIF1, Wnt inhibitory factor 1.

## References

[1] Clevers H. Wnt/beta-catenin signaling in development and disease. Cell (2006) 127(3):469–80. 10.1016/j.cell.2006.10.018 17081971

[2] Clevers H Nusse R Wnt/beta-catenin signaling and disease. Cell (2012) 149(6):1192–205. 10.1016/j.cell.2012.05.012 22682243

[3] Tak PP Firestein GS NF-kappaB: a key role in inflammatory diseases. J Clin Invest (2001) 107(1):7–11. 10.1172/JCI11830 11134171 PMC198552

[4] Li Q Verma IM NF-kappaB regulation in the immune system. Nat Rev Immunol (2002) 2(10):725–34. 10.1038/nri910 12360211

[5] Nejak-Bowen K Kikuchi A Monga SP Beta-catenin-NF-kappaB interactions in murine hepatocytes: a complex to die for. Hepatology (2013) 57(2):763–74. 10.1002/hep.26042 22941935 PMC3566301

[6] Karin M. NF-kappaB as a critical link between inflammation and cancer. Cold Spring Harb Perspect Biol (2009) 1(5):a000141. 10.1101/cshperspect.a000141 20066113 PMC2773649

[7] Ben-Neriah Y Karin M Inflammation meets cancer, with NF-kappaB as the matchmaker. Nat Immunol (2011) 12(8):715–23. 10.1038/ni.2060 21772280

[8] Pasparakis M. Regulation of tissue homeostasis by NF-kappaB signalling: implications for inflammatory diseases. Nat Rev Immunol (2009) 9(11):778–88. 10.1038/nri2655 19855404

[9] Anastas JN Moon RT. WNT signalling pathways as therapeutic targets in cancer. Nat Rev Cancer (2013) 13(1):11–26. 10.1038/nrc3419 23258168

[10] van Amerongen R Nusse R. Towards an integrated view of Wnt signaling in development. Development (2009) 136(19):3205–14. 10.1242/dev.033910 19736321

[11] Brembeck FH Rosario M Birchmeier W. Balancing cell adhesion and Wnt signaling, the key role of beta-catenin. Curr Opin Genet Dev (2006) 16(1):51–9. 10.1016/j.gde.2005.12.007 16377174

[12] Arce L Yokoyama NN Waterman ML. Diversity of LEF/TCF action in development and disease. Oncogene (2006) 25(57):7492–504. 10.1038/sj.onc.1210056 17143293

[13] Hoverter NP Waterman ML. A Wnt-fall for gene regulation: repression. Sci Signal (2008) 1(39):e43. 10.1126/scisignal.139pe43 18827220

[14] Hecht A Vleminckx K Stemmler MP van Roy F Kemler R. The p300/CBP acetyltransferases function as transcriptional coactivators of beta-catenin in vertebrates. EMBO J (2000) 19(8):1839–50. 10.1093/emboj/19.8.1839 10775268 PMC302022

[15] Idogawa M Yamada T Honda K Sato S Imai K Hirohashi S. Poly(ADP-ribose) polymerase-1 is a component of the oncogenic T-cell factor-4/beta-catenin complex. Gastroenterology (2005) 128(7):1919–36. 10.1053/j.gastro.2005.03.007 15940627

[16] Chiurillo MA Role of the Wnt/beta-catenin pathway in gastric cancer: an in-depth literature review. World J Exp Med (2015) 5(2):84–102. 10.5493/wjem.v5.i2.84 25992323 PMC4436943

[17] Stewart DJ. Wnt signaling pathway in non-small cell lung cancer. J Natl Cancer Inst (2014) 106(1):djt356. 10.1093/jnci/djt356 24309006

[18] Deng J Miller SA Wang HY Xia W Wen Y Zhou BP Beta-catenin interacts with and inhibits NF-kappa B in human colon and breast cancer. Cancer Cell (2002) 2(4):323–34. 10.1016/S1535-6108(02)00154-X 12398896

[19] Jian H Shen X Liu I Semenov M He X Wang XF. Smad3-dependent nuclear translocation of beta-catenin is required for TGF-beta1-induced proliferation of bone marrow-derived adult human mesenchymal stem cells. Genes Dev (2006) 20(6):666–74. 10.1101/gad.1388806 16543220 PMC1413283

[20] Rodilla V Villanueva A Obrador-Hevia A Robert-Moreno A Fernandez-Majada V Grilli A Jagged1 is the pathological link between Wnt and Notch pathways in colorectal cancer. Proc Natl Acad Sci U S A (2009) 106(15):6315–20. 10.1073/pnas.0813221106 19325125 PMC2669348

[21] Essers MA de Vries-Smits LM Barker N Polderman PE Burgering BM Korswagen HC. Functional interaction between beta-catenin and FOXO in oxidative stress signaling. Science (2005) 308(5725):1181–4. 10.1126/science.1109083 15905404

[22] Mazumdar J O’Brien WT Johnson RS LaManna JC Chavez JC Klein PS O2 regulates stem cells through Wnt/beta-catenin signalling. Nat Cell Biol (2010) 12(10):1007–13. 10.1038/ncb2102 20852629 PMC3144143

[23] Mitchell S Vargas J Hoffmann A Signaling via the NFkappaB system. Wiley Interdiscip Rev Syst Biol Med (2016) 8(3):227–41. 10.1002/wsbm.1331 26990581 PMC8363188

[24] Kingeter LM Paul S Maynard SK Cartwright NG Schaefer BC. Cutting edge: TCR ligation triggers digital activation of NF-kappaB. J Immunol (2010) 185(8):4520–4. 10.4049/jimmunol.1001051 20855880 PMC2950878

[25] Gerritsen ME Williams AJ Neish AS Moore S Shi Y Collins T. CREB-binding protein/p300 are transcriptional coactivators of p65. Proc Natl Acad Sci U S A (1997) 94(7):2927–32. 10.1073/pnas.94.7.2927 9096323 PMC20299

[26] Hassa PO Buerki C Lombardi C Imhof R Hottiger MO. Transcriptional coactivation of nuclear factor-kappaB-dependent gene expression by p300 is regulated by poly(ADP)-ribose polymerase-1. J Biol Chem (2003) 278(46):45145–53. 10.1074/jbc.M307957200 12960163

[27] Du Q Zhang X Cardinal J Cao Z Guo Z Shao L Wnt/beta-catenin signaling regulates cytokine-induced human inducible nitric oxide synthase expression by inhibiting nuclear factor-kappaB activation in cancer cells. Cancer Res (2009) 69(9):3764–71. 10.1158/0008-5472.CAN-09-0014 19383900

[28] Moreau M Mourah S Dosquet C Beta-catenin and NF-kappaB cooperate to regulate the uPA/uPAR system in cancer cells. Int J Cancer (2011) 128(6):1280–92. 10.1002/ijc.25455 20473943

[29] Rodriguez-Pinilla M Rodriguez-Peralto JL Hitt R Sanchez JJ Sanchez-Verde L Alameda F Beta-catenin, Nf-kappaB and FAS protein expression are independent events in head and neck cancer: study of their association with clinical parameters. Cancer Lett (2005) 230(1):141–8. 10.1016/j.canlet.2004.12.045 16253770

[30] Liu J Liao Y Ma K Wang Y Zhang G Yang R PI3K is required for the physical interaction and functional inhibition of NF-kappaB by beta-catenin in colorectal cancer cells. Biochem Biophys Res Commun (2013) 434(4):760–6. 10.1016/j.bbrc.2013.03.135 23583404

[31] Ma B van Blitterswijk CA Karperien M A Wnt/beta-catenin negative feedback loop inhibits interleukin-1-induced matrix metalloproteinase expression in human articular chondrocytes. Arthritis Rheum (2012) 64(8):2589–600. 10.1002/art.34425 22328140

[32] Ma B Fey M Hottiger MO WNT/beta-catenin signaling inhibits CBP-mediated RelA acetylation and expression of proinflammatory NF-kappaB target genes. J Cell Sci (2015) 128(14):2430–6. 10.1242/jcs.168542 26021349

[33] Sun J Hobert ME Duan Y Rao AS He TC Chang EB Crosstalk between NF-kappaB and beta-catenin pathways in bacterial-colonized intestinal epithelial cells. Am J Physiol Gastrointest Liver Physiol (2005) 289(1):G129–37. 10.1152/ajpgi.00515.2004 15790758

[34] Duan Y Liao AP Kuppireddi S Ye Z Ciancio MJ Sun J. Beta-catenin activity negatively regulates bacteria-induced inflammation. Lab Invest (2007) 87(6):613–24. 10.1038/labinvest.3700545 17384665

[35] Die L Yan P Jun Jiang Z Min Hua T Cai W Xing L. Glycogen synthase kinase-3 beta inhibitor suppresses *Porphyromonas gingivalis* lipopolysaccharide-induced CD40 expression by inhibiting nuclear factor-kappa B activation in mouse osteoblasts. Mol Immunol (2012) 52(1):38–49. 10.1016/j.molimm.2012.04.005 22580404

[36] Jiang R Chen D Hou J Tan Z Wang Y Huang X Survival and inflammation promotion effect of PTPRO in fulminant hepatitis is associated with NF-kappaB activation. J Immunol (2014) 193(10):5161–70. 10.4049/jimmunol.1303354 25339662

[37] Kim SJ Lim JY Lee JN Choe SK Kim YI Song SR Activation of beta-catenin by inhibitors of glycogen synthase kinase-3 ameliorates cisplatin-induced cytotoxicity and pro-inflammatory cytokine expression in HEI-OC1 cells. Toxicology (2014) 320:74–82. 10.1016/j.tox.2014.01.013 24560772

[38] Hao HP Wen LB Li JR Wang Y Ni B Wang R LiCl inhibits PRRSV infection by enhancing Wnt/beta-catenin pathway and suppressing inflammatory responses. Antiviral Res (2015) 117:99–109. 10.1016/j.antiviral.2015.02.010 25746333

[39] Kim JH Kim B Cai L Choi HJ Ohgi KA Tran C Transcriptional regulation of a metastasis suppressor gene by Tip60 and beta-catenin complexes. Nature (2005) 434(7035):921–6. 10.1038/nature03452 15829968

[40] Ke B Shen XD Kamo N Ji H Yue S Gao F Beta-catenin regulates innate and adaptive immunity in mouse liver ischemia-reperfusion injury. Hepatology (2013) 57(3):1203–14. 10.1002/hep.26100 23081841 PMC3594407

[41] Manicassamy S Reizis B Ravindran R Nakaya H Salazar-Gonzalez RM Wang YC Activation of beta-catenin in dendritic cells regulates immunity versus tolerance in the intestine. Science (2010) 329(5993):849–53. 10.1126/science.1188510 20705860 PMC3732486

[42] Winston JT Strack P Beer-Romero P Chu CY Elledge SJ Harper JW. The SCFbeta-TRCP-ubiquitin ligase complex associates specifically with phosphorylated destruction motifs in IkappaBalpha and beta-catenin and stimulates IkappaBalpha ubiquitination in vitro. Genes Dev (1999) 13(3):270–83. 10.1101/gad.13.3.270 9990852 PMC316433

[43] Spiegelman VS Slaga TJ Pagano M Minamoto T Ronai Z Fuchs SY. Wnt/beta-catenin signaling induces the expression and activity of betaTrCP ubiquitin ligase receptor. Mol Cell (2000) 5(5):877–82. 10.1016/S1097-2765(00)80327-5 10882123

[44] Wang X Adhikari N Li Q Guan Z Hall JL. The role of [beta]-transducin repeat-containing protein ([beta]-TrCP) in the regulation of NF-[kappa]B in vascular smooth muscle cells. Arterioscler Thromb Vasc Biol (2004) 24(1):85–90. 10.1161/01.ATV.0000104012.40720.c4 14592850

[45] Noubissi FK Elcheva I Bhatia N Shakoori A Ougolkov A Liu J CRD-BP mediates stabilization of betaTrCP1 and c-myc mRNA in response to beta-catenin signalling. Nature (2006) 441(7095):898–901. 10.1038/nature04839 16778892

[46] Ougolkov A Zhang B Yamashita K Bilim V Mai M Fuchs SY Associations among beta-TrCP, an E3 ubiquitin ligase receptor, beta-catenin, and NF-kappaB in colorectal cancer. J Natl Cancer Inst (2004) 96(15):1161–70. 10.1093/jnci/djh219 15292388

[47] Jia D Yang W Li L Liu H Tan Y Ooi S Beta-catenin and NF-kappaB co-activation triggered by TLR3 stimulation facilitates stem cell-like phenotypes in breast cancer. Cell Death Differ (2015) 22(2):298–310. 10.1038/cdd.2014.145 25257174 PMC4291491

[48] Bognar MK Vincendeau M Erdmann T Seeholzer T Grau M Linnemann JR Oncogenic CARMA1 couples NF-kappaB and beta-catenin signaling in diffuse large B-cell lymphomas. Oncogene (2016) 35(32):4269–81. 10.1038/onc.2015.493 26776161 PMC4981874

[49] Choi YS Hur J Jeong S. Beta-catenin binds to the downstream region and regulates the expression C-reactive protein gene. Nucleic Acids Res (2007) 35(16):5511–9. 10.1093/nar/gkm547 17704137 PMC2018623

[50] Yun K So JS Jash A Im SH Lymphoid enhancer binding factor 1 regulates transcription through gene looping. J Immunol (2009) 183(8):5129–37. 10.4049/jimmunol.0802744 19783677

[51] Anson M Crain-Denoyelle AM Baud V Chereau F Gougelet A Terris B Oncogenic beta-catenin triggers an inflammatory response that determines the aggressiveness of hepatocellular carcinoma in mice. J Clin Invest (2012) 122(2):586–99. 10.1172/JCI43937 22251704 PMC3266772

[52] Phelps RA Broadbent TJ Stafforini DM Jones DA. New perspectives on APC control of cell fate and proliferation in colorectal cancer. Cell Cycle (2009) 8(16):2549–56. 10.4161/cc.8.16.9278 19597346

[53] Kuphal S Poser I Jobin C Hellerbrand C Bosserhoff AK. Loss of E-cadherin leads to upregulation of NFkappaB activity in malignant melanoma. Oncogene (2004) 23(52):8509–19. 10.1038/sj.onc.1207831 15378016

[54] Bandino A Compagnone A Bravoco V Cravanzola C Lomartire A Rossetto C Beta-catenin triggers nuclear factor kappaB-dependent up-regulation of hepatocyte inducible nitric oxide synthase. Int J Biochem Cell Biol (2008) 40(9):1861–71. 10.1016/j.biocel.2008.01.029 18343708

[55] Solanas G Porta-de-la-Riva M Agusti C Casagolda D Sanchez-Aguilera F Larriba MJ E-cadherin controls beta-catenin and NF-kappaB transcriptional activity in mesenchymal gene expression. J Cell Sci (2008) 121(Pt 13):2224–34. 10.1242/jcs.021667 18565826

[56] Zhao W Sun Z Wang S Li Z Zheng L. Wnt1 participates in inflammation induced by lipopolysaccharide through upregulating scavenger receptor A and NF-kB. Inflammation (2015) 38(4):1700–6. 10.1007/s10753-015-0147-8 25749569 PMC4495710

[57] Schon S Flierman I Ofner A Stahringer A Holdt LM Kolligs FT Beta-catenin regulates NF-kappaB activity via TNFRSF19 in colorectal cancer cells. Int J Cancer (2014) 135(8):1800–11. 10.1002/ijc.28839 24623448

[58] Jang J Ha JH Chung SI Yoon Y Beta-catenin regulates NF-kappaB activity and inflammatory cytokine expression in bronchial epithelial cells treated with lipopolysaccharide. Int J Mol Med (2014) 34(2):632–8. 10.3892/ijmm.2014.1807 24938929

[59] Ma B Zhong L van Blitterswijk CA Post JN Karperien M T cell factor 4 is a pro-catabolic and apoptotic factor in human articular chondrocytes by potentiating nuclear factor kappaB signaling. J Biol Chem (2013) 288(24):17552–8. 10.1074/jbc.M113.453985 23603903 PMC3682554

[60] Cummins EP Berra E Comerford KM Ginouves A Fitzgerald KT Seeballuck F Prolyl hydroxylase-1 negatively regulates IkappaB kinase-beta, giving insight into hypoxia-induced NFkappaB activity. Proc Natl Acad Sci U S A (2006) 103(48):18154–9. 10.1073/pnas.0602235103 17114296 PMC1643842

[61] Scholz CC Cavadas MA Tambuwala MM Hams E Rodriguez J von Kriegsheim A Regulation of IL-1beta-induced NF-kappaB by hydroxylases links key hypoxic and inflammatory signaling pathways. Proc Natl Acad Sci U S A (2013) 110(46):18490–5. 10.1073/pnas.1309718110 24145445 PMC3832034

[62] Saegusa M Hashimura M Kuwata T Hamano M Okayasu I. Crosstalk between NF-kappaB/p65 and beta-catenin/TCF4/p300 signalling pathways through alterations in GSK-3beta expression during trans-differentiation of endometrial carcinoma cells. J Pathol (2007) 213(1):35–45. 10.1002/path.2198 17607667

[63] Buss H Dorrie A Schmitz ML Frank R Livingstone M Resch K Phosphorylation of serine 468 by GSK-3beta negatively regulates basal p65 NF-kappaB activity. J Biol Chem (2004) 279(48):49571–4. 10.1074/jbc.C400442200 15465828

[64] Hoeflich KP Luo J Rubie EA Tsao MS Jin O Woodgett JR. Requirement for glycogen synthase kinase-3beta in cell survival and NF-kappaB activation. Nature (2000) 406(6791):86–90. 10.1038/35017574 10894547

[65] Martin M Rehani K Jope RS Michalek SM. Toll-like receptor-mediated cytokine production is differentially regulated by glycogen synthase kinase 3. Nat Immunol (2005) 6(8):777–84. 10.1038/ni1221 16007092 PMC1933525

[66] Kotliarova S Pastorino S Kovell LC Kotliarov Y Song H Zhang W Glycogen synthase kinase-3 inhibition induces glioma cell death through c-MYC, nuclear factor-kappaB, and glucose regulation. Cancer Res (2008) 68(16):6643–51. 10.1158/0008-5472.CAN-08-0850 18701488 PMC2585745

[67] Hofmann C Dunger N Scholmerich J Falk W Obermeier F Glycogen synthase kinase 3-beta: a master regulator of toll-like receptor-mediated chronic intestinal inflammation. Inflamm Bowel Dis (2010) 16(11):1850–8. 10.1002/ibd.21294 20848477

[68] Thyssen G Li TH Lehmann L Zhuo M Sharma M Sun Z. LZTS2 is a novel beta-catenin-interacting protein and regulates the nuclear export of beta-catenin. Mol Cell Biol (2006) 26(23):8857–67. 10.1128/MCB.01031-06 17000760 PMC1636836

[69] Cho HH Song JS Yu JM Yu SS Choi SJ Kim DH Differential effect of NF-kappaB activity on beta-catenin/Tcf pathway in various cancer cells. FEBS Lett (2008) 582(5):616–22. 10.1016/j.febslet.2008.01.029 18242184

[70] Chang J Liu F Lee M Wu B Ting K Zara JN NF-kappaB inhibits osteogenic differentiation of mesenchymal stem cells by promoting beta-catenin degradation. Proc Natl Acad Sci U S A (2013) 110(23):9469–74. 10.1073/pnas.1300532110 23690607 PMC3677422

[71] Gwak J Park S Cho M Song T Cha SH Kim DE *Polysiphonia japonica* extract suppresses the Wnt/beta-catenin pathway in colon cancer cells by activation of NF-kappaB. Int J Mol Med (2006) 17(6):1005–10. 10.3892/ijmm.17.6.1005 16685408

[72] Lamberti C Lin KM Yamamoto Y Verma U Verma IM Byers S Regulation of beta-catenin function by the IkappaB kinases. J Biol Chem (2001) 276(45):42276–86. 10.1074/jbc.M104227200 11527961

[73] Albanese C Wu K D’Amico M Jarrett C Joyce D Hughes J IKKalpha regulates mitogenic signaling through transcriptional induction of cyclin D1 via Tcf. Mol Biol Cell (2003) 14(2):585–99. 10.1091/mbc.02-06-0101 12589056 PMC149994

[74] Carayol N Wang CY. IKKalpha stabilizes cytosolic beta-catenin by inhibiting both canonical and non-canonical degradation pathways. Cell Signal (2006) 18(11):1941–6. 10.1016/j.cellsig.2006.02.014 16616828

[75] Schwitalla S Fingerle AA Cammareri P Nebelsiek T Goktuna SI Ziegler PK Intestinal tumorigenesis initiated by dedifferentiation and acquisition of stem-cell-like properties. Cell (2013) 152(1–2):25–38. 10.1016/j.cell.2012.12.012 23273993

[76] Li D Beisswenger C Herr C Hellberg J Han G Zakharkina T Myeloid cell RelA/p65 promotes lung cancer proliferation through Wnt/beta-catenin signaling in murine and human tumor cells. Oncogene (2014) 33(10):1239–48. 10.1038/onc.2013.75 23563178

[77] Yun K Choi YD Nam JH Park Z Im SH NF-kappaB regulates Lef1 gene expression in chondrocytes. Biochem Biophys Res Commun (2007) 357(3):589–95. 10.1016/j.bbrc.2007.03.170 17445771

[78] Hyun Hwa C Hye Joon J Ji Sun S Yong Chan B Jin Sup J. Crossregulation of beta-catenin/Tcf pathway by NF-kappaB is mediated by lzts2 in human adipose tissue-derived mesenchymal stem cells. Biochim Biophys Acta (2008) 1783(3):419–28. 10.1016/j.bbamcr.2007.08.005 17950943

[79] Hanahan D Weinberg RA Hallmarks of cancer: the next generation. Cell (2011) 144(5):646–74. 10.1016/j.cell.2011.02.013 21376230

[80] Oguma K Oshima H Aoki M Uchio R Naka K Nakamura S Activated macrophages promote Wnt signalling through tumour necrosis factor-alpha in gastric tumour cells. EMBO J (2008) 27(12):1671–81. 10.1038/emboj.2008.105 18511911 PMC2413189

[81] Zhang Y Tomann P Andl T Gallant NM Huelsken J Jerchow B Reciprocal requirements for EDA/EDAR/NF-kappaB and Wnt/beta-catenin signaling pathways in hair follicle induction. Dev Cell (2009) 17(1):49–61. 10.1016/j.devcel.2009.05.011 19619491 PMC2859042

[82] Fliniaux I Mikkola ML Lefebvre S Thesleff I. Identification of dkk4 as a target of Eda-A1/Edar pathway reveals an unexpected role of ectodysplasin as inhibitor of Wnt signalling in ectodermal placodes. Dev Biol (2008) 320(1):60–71. 10.1016/j.ydbio.2008.04.023 18508042

[83] Wen F Liu Y Wang W Li M Guo F Sang Y Adenomatous polyposis coli genotype-dependent toll-like receptor 4 activity in colon cancer. Oncotarget (2016) 7(7):7761–72. 10.18632/oncotarget.6844 26760960 PMC4884952

